# Connexin Hemichannels Contribute to the Activation of cAMP Signaling Pathway and Renin Production

**DOI:** 10.3390/ijms21124462

**Published:** 2020-06-23

**Authors:** Jingru Hong, Jian Yao

**Affiliations:** Division of Molecular Signaling, Department of the Advanced Biomedical Research, Interdisciplinary Graduate School of Medicine, University of Yamanashi, Chuo 409-3898, Japan; lulujane0130@gmail.com

**Keywords:** connexin, hemichannel, Ca^2+^, renin, cAMP, renin-secreting cells

## Abstract

Connexin hemichannels play an important role in the control of cellular signaling and behaviors. Given that lowering extracellular Ca^2+^, a condition that activates hemichannels, is a well-characterized stimulator of renin in juxtaglomerular cells, we, therefore, tested a potential implication of hemichannels in the regulation of renin in As4.1 renin-secreting cells. Lowering extracellular Ca^2+^ induced hemichannel opening, which was associated with cAMP signaling pathway activation and increased renin production. Blockade of hemichannels with inhibitors or downregulation of Cxs with siRNAs abrogated the activation of cAMP pathway and the elevation of renin. Further analysis revealed that cAMP pathway activation was blocked by adenylyl cyclase inhibitor SQ 22536, suggesting an implication of adenyl cyclase. Furthermore, the participation of hemichannels in the activation of the cAMP signaling pathway was also observed in a renal tubular epithelial cell line NRK. Collectively, our results characterized the hemichannel opening as a presently unrecognized molecular event involved in low Ca^2+^-elicited activation of cAMP pathway and renin production. Our findings thus provide novel mechanistic insights into the low Ca^2+^-initiated cell responses. Given the importance of cAMP signaling pathway in the control of multiple cellular functions, our findings also highlight the importance of Cx-forming channels in various pathophysiological situations.

## 1. Introduction

The renin-angiotensin system is a major regulatory system controlling extracellular fluid volume and blood pressure. The rate-limiting enzyme in this hormonal cascade is renin, which is secreted into the circulation by renal juxtaglomerular (JG) cells of the afferent arteriole. Renin secretion is controlled by several factors, such as blood pressure, sodium chloride load, sympathetic nerves, hormones, cytokines, and vasoactive materials. Although the signal pathways underlying the actions of these factors differ, it is generally accepted that a change in intracellular Ca^2+^ is the common pathway through which renin secretion from JG cells is governed. In contrast to other secretory cells, in which an increase in Ca^2+^ usually promotes secretion, renin secretion is inversely related to the Ca^2+^ concentration in JG cells [1,2,3,4,5,6,7].

The molecular mechanisms by which Ca^2+^ regulates renin have been the focus of many previous investigations [1,2,4,5,8]. Accumulated evidences indicate that Ca^2+^ regulates renin through modulation of adenylyl cyclase activities. Low Ca^2+^ enhances the activities of the Ca^2+^-inhibitable isoforms of adenylyl cyclases in JG cells, thus increasing intracellular cyclic adenosine monophosphate (cAMP) and stimulating renin synthesis [1,2,8]. Oppositely, elevation in intracellular Ca^2+^ suppresses adenylyl cyclase and inhibits renin [5]. In fact, cAMP is a well-characterized secondary messenger in stimulation of renin synthesis and secretion [8,9,10,11]. cAMP-elevating chemicals such as prostaglandins, kinins, and alpha-adrenergic agonists, all stimulate renin, whereas agents that inhibit the cAMP pathway suppress renin [11,12,13].

Gap junctions (GJs) are intercellular channels that permit the direct exchange of ions, secondary messengers, and small signaling molecules among neighboring cells. Each GJ channel is composed of two hemichannels that reside in the plasma membrane of two closely apposed cells. Each hemichannel is composed of six protein subunits termed connexin (Cx). Up to now, more than 20 different Cx molecules have been identified. Gap junction-mediated intercellular communication (GJIC) plays an important role in the regulation of cell signaling and functions [14,15,16]. In the juxtaglomerular apparatus of kidney, GJIC provides a pathway for signal transduction and coordination of multicellular functions. Disruption of cell-to-cell communication in the JGA alters preglomerular vascular tone and renin secretion [15,17,18]. Knockout of Cx40 in renin-secreting cells led to increased renin level and renin-dependent hypertension, which was presumably due to dysfunction of gap junction-mediated negative regulatory signals [19,20,21].

Besides intercellular GJ channels, the non-junctional Cx hemichannels are also activated under various pathophysiological situations and participate in the regulation of cellular behaviors through the release of mediators such as cAMP, adenosine triphosphate (ATP), nicotinamide adenine dinucleotide (NAD^+^), glutamate or prostaglandin E2 (PGE2) [16,22,23,24,25]. Several considerations promoted us to speculate a potential implication of hemichannels in the control of renin. First, among the molecules released by hemichannels, some of them are closely related to cAMP signaling pathway such as cAMP, ATP and PGE2 [24,25,26]. Second, removing extracellular Ca^2+^, a situation that stimulates renin, also activates hemichannels [9,27,28,29,30,31]. Third, participation of several membrane channels in the regulation of renin has been previously documented [32,33,34]. Therefore, we designed this study to address the role of hemichannels in low Ca^2+^-induced activation of cAMP pathway and renin production.

Here, we present our results that hemichannel opening is requisite for the effective activation of the cAMP signaling pathway-induced low Ca^2+^ in mouse renin-secreting cells. Given the importance of the cAMP signaling pathway in multiple pathophysiological situations, the activation of the pathway by hemichannels provides a novel mechanistic insight into the actions of Cx hemichannels and suggests that Cx channels are critically involved in the control of many cellular processes.

## 2. Results

### 2.1. Removing Extracellular Ca^2+^ Activates Hemichannels in As4.1 Cells

**Figure 1:**
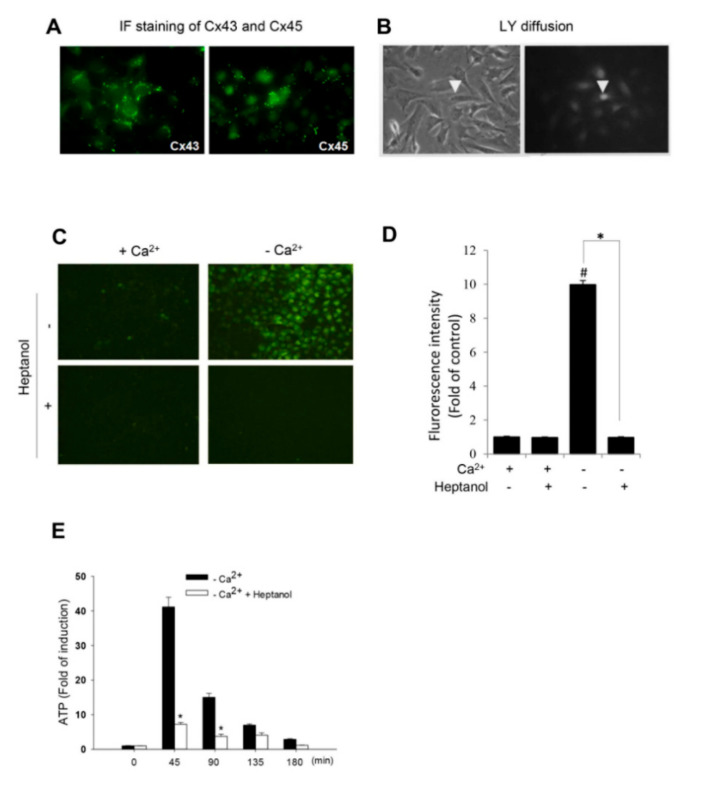
Induction of hemichannel opening by removal of extracellular Ca^2+^. (**A**) Immunofluorescent staining of Cx43 and Cx45 in As4.1 cells. As4.1 cells were stained for expression of Cx43 and Cx45. Note the localization of Cx43 and Cx45 in both membrane and cytoplasm. Magnification, X320. (**B**) Diffusion of LY following single cell injection. LY was injected into single As4.1 cell (arrow head) and the diffusion of LY from the injected cell to neighboring cells was photographed (right image). Magnification, X320. (**C**,**D**) Effects of extracellular Ca^2+^ depletion on hemichannels activities. (**C**) Effects of Ca^2+^ depletion on cellular uptake of LY. As4.1 cells were pretreated with or without 3 mM heptanol for 15 min. After that, they were exposed to either normal or Ca^2+^-free culture medium that contained 0.05% LY in the presence of the same concentrations of heptanol for an additional 20 min. The influx of LY was photographed (Magnification, X200). The fluorescent intensity of the cells in C was measured with ImageJ and expressed as relative unit against control (mean ± SE, *n* = 20; ^#^ *p* < 0.01 versus control, * *p* < 0.01). Data shown areone representative result out of four separate experiments. (**E**) Ca^2+^ depletion on extracellular release of ATP. As4.1 cells were exposed to Ca^2+^-free culture medium in the presence or absence of 3 mM heptanol for the indicated time intervals. Cell supernatants were collected and quantitated for ATP concentration. Results are expressed as fold induction relative to control (mean ± SE, *n* = 4). * *p* < 0.01 versus-Ca^2+^ control.

To determine hemichannel activity after removing the extracellular Ca^2+^, we have used a renin-expressing cell line As4.1. This cell line was isolated from a mouse renal tumor and has been well characterized for the synthesis, storage and secretion of renin. We first confirmed that As4.1 cells expressed functional Cx molecules. Based on the previous reports and availability of antibodies [35], we immunostained As4.1 cells with anti-Cx43 and anti-Cx45 antibodies. As shown Figure 1A, there was positive punctate staining of Cx43 and Cx45 around the perinuclear and cell-to-cell contact regions. Consistent with the membrane localization of Cxs, As4.1 cells were well coupled with functional GJs. Injection of fluorescent dye lucifer yellow (LY) into single cell led to diffusion of the dye from the injected cell to several neighboring cells (Figure 1B). These results thus indicate that As4.1 cells express functional Cxs.

We and others have previously reported that the removal of extracellular Ca^2+^ activated hemichannels [9,27,29,30,31]. We therefore tested whether this also occurred in As4.1 cells. Figure 1C–E show that the exposure of As4.1 cells to Ca^2+^-free medium initiated a rapid exchange of small molecules between the inside and outside of the plasma membrane, as indicated by the influx of LY and efflux of ATP. In the presence of the hemichannel inhibitor heptanol, this exchange was almost completely blocked. These results indicate that Ca^2+^ deprivation activates hemichannels in As4.1 cells.

### 2.2. Hemichannels Mediate Low Ca^2+^-Triggered Activation of cAMP Signaling Pathway

**Figure 2:**
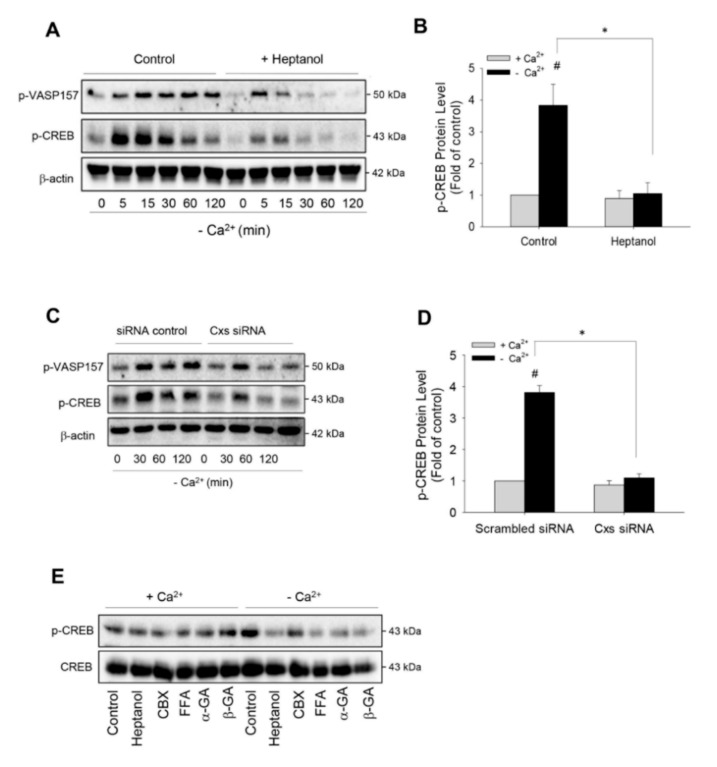
Effects of dysfunction of hemichannels on low Ca^2+^-induced activation of cAMP signaling pathway. (**A**) Effect of hemichannel inhibitor heptanol on PKA activation. As4.1 cells were pretreated with or without 3 mM heptanol for 30 min, then exposed to Ca^2+^-free medium in the presence or absence of heptanol for the indicated time intervals. Cellular proteins were extracted and subjected to Western blot analysis for the phosphorylated levels of VASP and CREB. β-actin was used as a loading control. (**B**) Desitometric quantitation of the phosphorylated level of CREB. The intensities of p-CREB signal at the time point of 30 min were measured using the Scion Image software. The data are expressed as relative intensity of the band against the control (mean ± SE, n = 4). ^#^ *p* < 0.01 versus normal Ca^2+^ control; * *p* < 0.01 versus heptanol-treated cells. (**C**) Effect of siRNA treatment on cAMP signaling pathway. As4.1 cells were treated with siRNA control or siRNA cocktail targeting against Cxs37, 40, 43 and 45 for 24 h and exposed to Ca^2+^-free medium for the indicated times. The levels of phosphorylated VASP157 and phosphorylated CREB were evaluated by Western blot analysis. (**D**) Densitometric quantitation of the phosphorylated level of CREB at 30 min point shown in C. The data are expressed as relative intensity of the band against the control (mean ± SE, *n* = 3). ^#^ *p* < 0.01 versus normal Ca^2+^ control; * *p* < 0.01 vs. siRNA-treated cells. (**E**) Effect of various gap junction inhibitors on low Ca^2+^-induced CREB phosphorylation. As4.1 cells were treated with 3 mM heptanol, 100 μM CBX, 50 μM FFA, 10 μM α-or β-GA in the way same as A and analyzed for the phosphorylated and total level of CREB.

To determine the role of hemichannels in Ca^2+^ deprivation-induced renin production, we first examined the influence of hemichannels on renin-regulating cAMP pathway [2,9,11]. Figure 2A shows that the removal of extracellular Ca^2+^ elevated vasodilator-stimulated phosphoprotein (VASP) and cAMP response element binding protein (CREB) phosphorylation, two validated substrates of cAMP-dependent protein kinase A (PKA) [9,36]. This effect was rapid, being observable as early as 5 min and lasted for at least 120 min. Intriguingly, in the presence of heptanol, the elevation of phosphorylated VASP and CREB was markedly suppressed. This suppression was more pronounced at the later time points. Heptanol almost completely abolished CREB phosphorylation at 30 min (Figure 2A,B).

To confirm that the blocking effect of heptanol was due to its action on the Cx-forming channel, we have downregulated Cxs using siRNA cocktail targeting against Cx37, Cx40, Cx43 and Cx45, which are reported to be expressed in As4.1 cells [35]. Figure 2C,D show that siRNA treatment prevented the phosphorylation of PKA substrates, VASP and CREB, in a way similar to heptanol. In addition, besides heptanol, other hemichannel inhibitors, such as CBX, FFA, α and β-GA also effectively inhibited low-Ca^2+^-initiated CREB phosphorylation (Figure 2E). These results indicate that Cx-forming hemichannels contribute to the low Ca^2+^-elicited activation of the cAMP pathway.

### 2.3. Low Ca^2+^-Induced Activation of cAMP Signaling is Mediated by Adenylyl Cyclase

**Figure 3:**
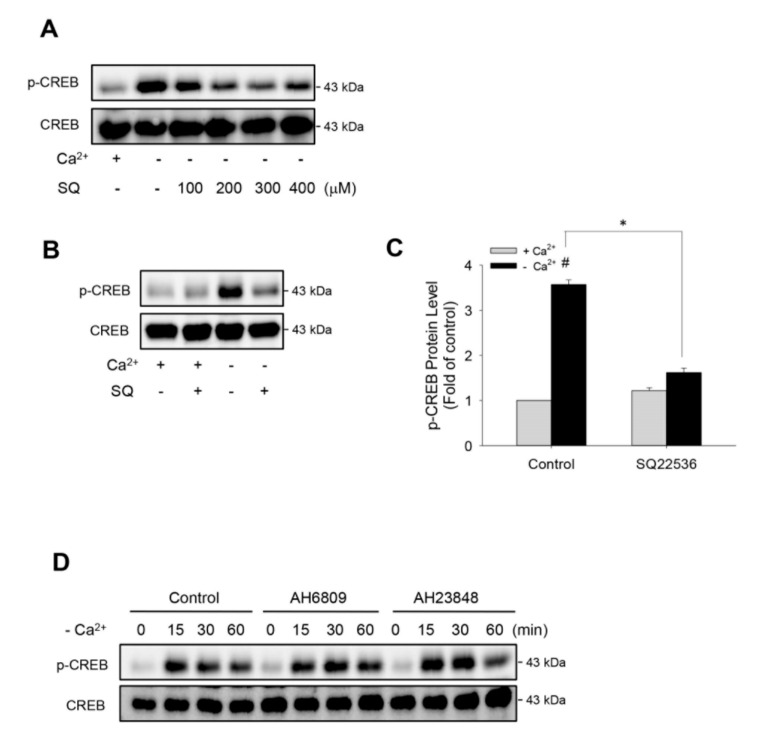
Effects of adenylyl cyclase inhibitor and prostaglandin receptor blockers on low Ca^2+^-induced CREB phosphorylation. (**A**,**B**) Suppression of CREB phosphorylation by adenylyl cyclase inhibitor. As4.1 cells were pretreated with the indicated concentrations of SQ 22536 (**A**) or 300 μM SQ 22536 (**B**) for 30 min, then exposed to Ca^2+^-free medium for an additional 15 min. Cellular proteins were extracted and subjected to Western blot analysis for phosphorylated and total level of CREB. (**C**) Densitometric quantitation of the phosphorylated level of CREB in B. The data are expressed as relative intensity of the band against the control (mean ± SE, *n* = 4). ^#^ *p* < 0.01 versus normal Ca^2+^ control; * *p* < 0.01 versus SQ 22536-treated cells. (**D**) Effect of EP2 and EP4 blockers on CREB phosphorylation. As4.1 cells were pretreated with or without 20 μM EP2 inhibitor AH6809 or EP4 inhibitor AH23848 for 30 min, then exposed to Ca^2+^-free medium for the indicated time intervals. Cellular proteins were extracted and subjected to Western blot analysis for phosphorylated and total level of CREB.

Several reports described that low Ca^2+^-induced renin secretion was mediated by Ca^2+^-inhibitable adenylyl cyclase [1,2]. We therefore tested its involvement under our experimental settings. As shown in Figure 3A, the induction of CREB phosphorylation following the removal of Ca^2+^ was blocked by adenylyl cyclase inhibitor SQ 22536 in a concentration-dependent manner. A high concentration of SQ 22536 completely abolished the phosphorylation of CREB (Figure 3B,C).

As hemichannel-derived PGE_2_ activates adenylyl cyclase [10,24,37], we hence examined the potential role of PGE_2_. To this end, we have blocked the receptors that mediate cAMP-elevating effect of PGE_2_ [10,37]. Figure 3D shows that blockade of EP2 and EP4 with AH6809 and AH23848 failed to affect CREB phosphorylation. These observations indicate that low Ca^2+^ activates adenylyl cyclase through the PGE_2_-independent signaling mechanism.

### 2.4. Hemichannels Contribute to Low Ca^2+^-Elicited Expression of Renin

**Figure 4:**
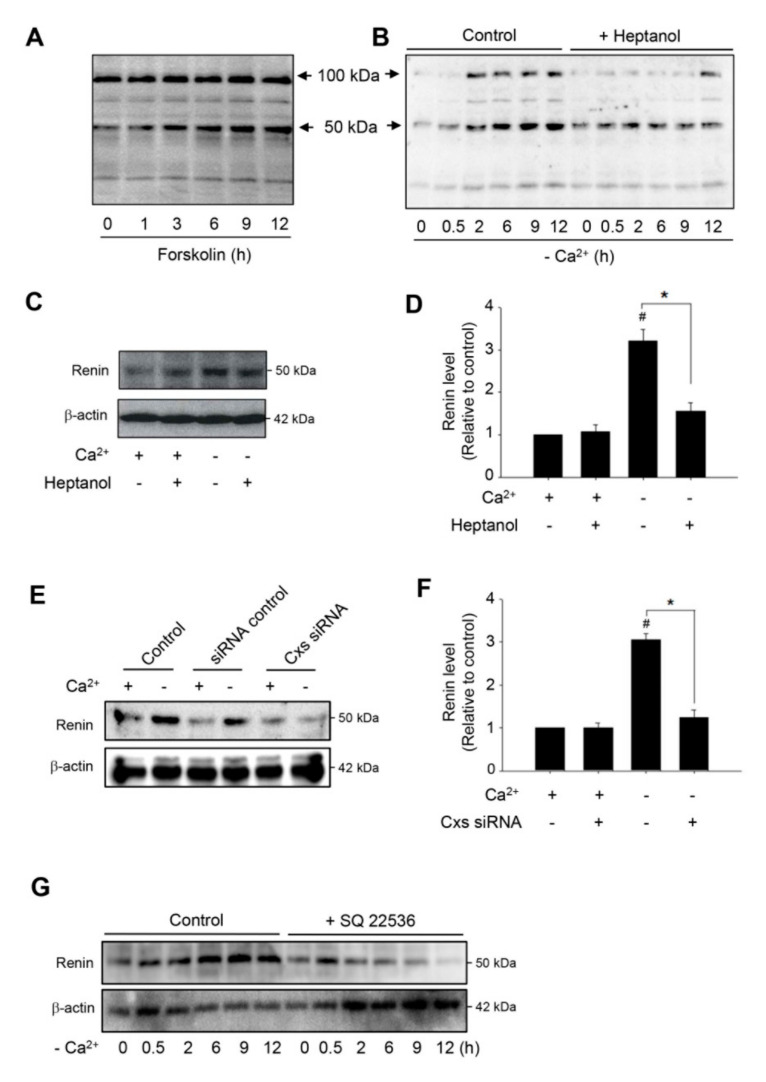
Effects of dysfunction of hemichannels on renin protein levels. (**A**) Induction of renin production by adenyl cyclase activator forskolin. (**B**–**D**). Effect of hemichannel inhibitor heptanol on renin protein levels. As4.1 cells were pretreated with 3 mM heptanol for 30 min, then treated with Ca^2+^-free medium with or without the same amount of heptanol for the indicated times (**B**) or 12 h (**C**). Cellular proteins were examined by Western blot with an anti-renin antibody. (**D**) The densitometric quantitation of the intensities of renin signal in (**C**). The data are expressed as relative intensity of the band against the control (mean ± SE, *n* = 4). ^#^ *p* < 0.01 versus normal Ca^2+^ control; * *p* < 0.01 versus heptanol-treated cells. (**E**) Downregulation of Cxs with siRNA on renin protein levels. As4.1 cells were transfected with siRNA control or SiRNA cocktail targeting against Cx37, 40, 43 and 45 for 24 h, and then exposed to Ca^2+^-free medium for an additional 12 h. Cellular protein was extracted and subjected to Western blot analysis for renin. Expression of β-actin is shown as a loading control. (**F**) Densitometric quantitation of renin levels in D. The data are expressed as relative intensity of the band against the control (mean ± SE, *n* = 3). ^#^ *p* < 0.01 versus normal Ca^2+^ control; * *p* < 0.01 versus siRNA-treated cells. (**G**) Influence of blockade of adenylyl cyclase on renin protein levels. As4.1 cells were pretreated with 300 μM adenylyl cyclase inhibitor SQ 22536 for 30 min, then treated with Ca^2+^-free medium with or without the same amount of SQ 22536 for the indicated times intervals. Cellular proteins were subjected to Western blot analysis for renin.

cAMP is the major second messenger involved in the control of renin production [9,11,38]. In this study, we observed that the activation of the cAMP pathway in As4.1 cells with adenyl cyclase activator forskolin resulted in a time-dependent elevation in renin protein level at the MW of 50 kDa (Figure 4A). Intriguingly, removing extracellular Ca^2+^ also achieved the same effect (Figure 4B). Furthermore, consistent with the mediating role of hemichannels on cAMP activation, inhibition of hemichannels with heptanol or siRNA significantly blunted the elevation (Figure 4B–F). In addition, the inhibition of adenylyl cyclase with SQ 22536 also abolished the elevation (Figure 4G). Collectively, these results indicate that hemichannels contribute to low Ca^2+^-elicited renin production through its action on the cAMP signaling pathway.

### 2.5. Hemichannels Mediate Low Ca^2+^-Elicited Activation of cAMP Signaling Pathway in Rat Renal Tubular Epithelial Cell NRK

**Figure 5:**
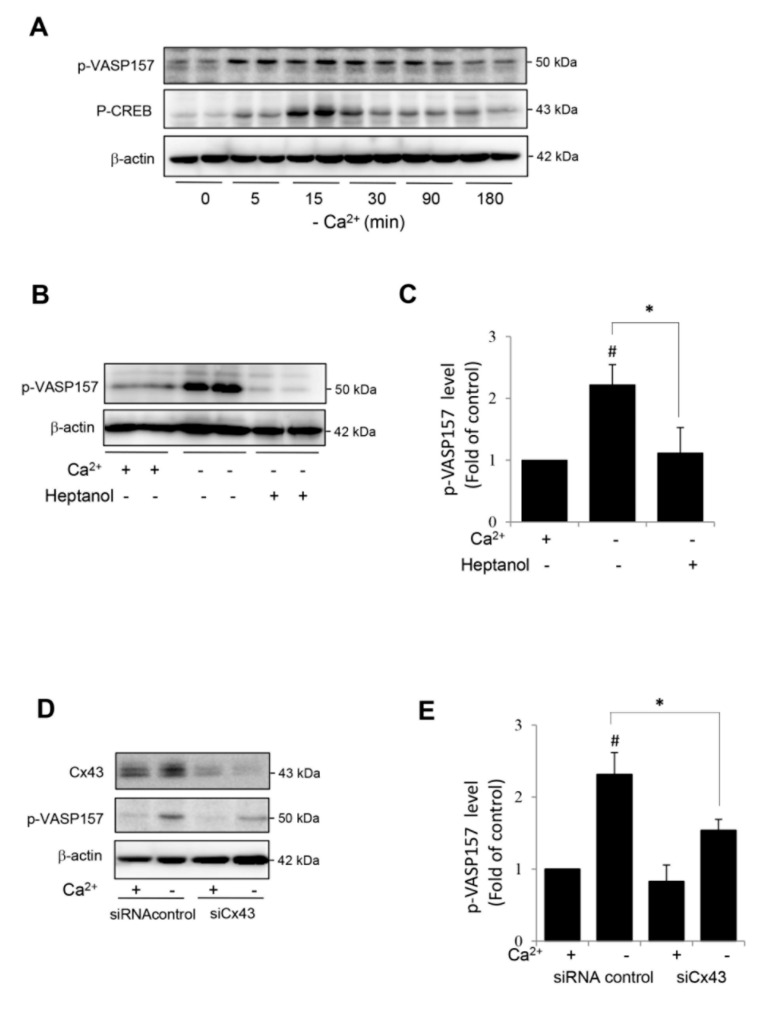
Hemichannel-mediated activation of cAMP signaling pathway in renal tubular epithelial NRK cells. (**A**) Induction of VASP and CREB phosphorylation by Ca^2+^ deprivation in NRK cells. NRK cells were exposed to Ca2^+^-free medium for the indicated time intervals. Cellular proteins were extracted and subjected to Western blot analysis for phosphorylated level of VASP and CREB. β-actin was used as a loading control. (**B**–**E**) Effect of hemichannel inhibitor heptanol (**B**,**C**) and Cx43 siRNA treatment (**D**,**E**) on PKA activation. NRK cells were pretreated with 3 mM heptanol for 30 min (**B**) or Cx43 siRNA for 48 h (**D**) before exposing to Ca^2+^-free medium for 10 min. Cellular proteins were extracted and subjected to Western blot analysis for phosphorylated level of VASP and β-actin. (**C**,**E**) Densitometric quantitation of the phosphorylated level of VASP in B and D, respectively. The intensities of p-VASP signal were measured by using the Scion Image software. The data are expressed as relative intensity of the band against the control (mean ± SE, *n* = 4). ^#^ *p* < 0.05 versus normal Ca^2+^ control; * *p* < 0.05 versus heptanol or siRNA-treated cells.

We also tested whether the hemichannel-mediated activation of the cAMP signaling pathway could also occur in other cell types. For this purpose, we have used a rat renal tubular epithelial cell line NRK-52E, which was developed from an epithelioid clone of mixed culture of normal rat kidney cells. In our previous study, we have demonstrated that Cx43 was the predominant connexin in NRK cells and that the deprivation of extracellular Ca^2+^ also activated hemichannels in NRK cells [29,30,31,39]. In consistence with the findings in renin-secreting cells, Ca^2+^ deprivation also activated the cAMP signaling pathway in NRK, as evidenced by the elevated phosphorylation of VASP and CREB (Figure 5A). This elevation was mediated by Cx43 hemichannels. The inhibition of hemichannels with heptanol or downregulation of Cx43 with siRNA significantly blunted VASP phosphorylation (Figure 5B–E). These results indicate that the hemichannel-mediated activation of the cAMP signaling pathway is not cell type-specific.

## 3. Discussion

Using a well-characterized mouse renin-secreting cell line As4.1 cells, we characterized activation of Cx hemichannels as a key molecular event implicated in the low Ca^2+^-induced activation of the cAMP pathway and the induction of renin. Given the importance of both cAMP signaling and hemichannels in the control of many cellular responses, our finding could have important implications.

cAMP pathway is a well-characterized signaling mechanism for the stimulation of renin synthesis and secretion [6,8,9,40]. The pathway starts from the activation of adenylyl cyclase that catalyzes the conversion of ATP into cAMP. The accumulated cAMP activates PKA, which phosphorylates several proteins and regulates a wide spectrum of cell behaviors. It has been reported that elevated cAMP stimulates renin gene expression in JG cells by phosphorylation of CREB through PKA [9,11]. Consistent with these reports, the elevation in renin in this study was preceded by CREB phosphorylation. The prevention of CREB phosphorylation by the inhibition of adenylyl cyclase blunted renin elevation. These observations support the notion that low Ca^2+^ stimulates renin through the cAMP signaling pathway [6,8,9,40].

One of the intriguing findings in this study is that dysfunction of hemichannels completely blunted the Ca^2+^ deprivation-induced activation of the cAMP signaling pathway. Activation of hemichannels by removing extracellular Ca^2+^ has been previously demonstrated at both structural and functional levels. Using an atomic force microscope, Muller et al. observed that lowing extracellular Ca^2+^ increased outer hemichannel pore diameter [9]. Consistently, many investigators observed increased hemichannel permeability [22,23,27,29,30,31]. In agreement with these reports, we detected a rapid exchange of small molecules between the inside and the outside of the cell membrane following the elimination of extracellular Ca^2+^ in As4.1 cells. Disruption of the exchange abolished the activation of the cAMP pathway. Our results thus pointed to a critical role of hemichannels in cell responses to low Ca^2+^ stimulation.

The question naturally occurs as to how hemichannels regulated the cAMP signaling pathway. Consistent with previous reports [1,2,8], the low Ca^2+^-induced activation of the cAMP pathway in As4.1 cells was mediated by adenylyl cyclase. Inhibition of adenylyl cyclase with SQ22536 completely prevented CREB phosphorylation. In this context, it was conceivable that hemichannels might affect the cAMP pathway through the modulation of adenylyl cyclase activities. Indeed, several hemichannel-derived mediators, such as PGE2 and ATP, have been reported to be able to activate adenylyl cyclase [10,11,37,41,42]. However, our data failed to support an involvement of PGE2 because blockade of the relevant PGE_2_ receptors did not affect CREB phosphorylation. As for the role of ATP on the activation of the cAMP pathway, there are also conflicting reports. A recent study by Iwamoto, et al. demonstrated that efflux of ATP through pannexin hemichannel caused a reduction in intracellular cAMP [43]. Furthermore, ATP is known to induce intra- and inter-cellular Ca^2+^ signaling, which might negatively affect the cAMP pathway and renin production [7]. Therefore, the mechanisms by which hemichannels regulated cAMP pathway remain to be clarified. Of note, hemichannels might affect cAMP signaling pathway through channel-mediated intra- and extra-cellular exchange of ions, such as Ca^2+^, chloride and the uncharacterized cations as well. Implication of these ions in the regulation of renin has been previously described [8,33]. This speculation also needs to be tested in the future.

By using RT-PCR, Ryan, et al. found that As4.1 cells express several isoforms of Cx molecules, including Cx37, Cx40, Cx43 and Cx45 [35]. Consistently, we confirmed the presence of Cx43 and Cx45 in As4.1 cells at protein level by IF staining and Western blot analysis. In addition, we also detected a positive band of Cx40 in Western blot analysis (data not shown). To completely block hemichannels, we have used siRNA targeting against all reported Cxs, including Cx37, 40, 43 and 45. This strategy achieved a suppressive effect comparable to heptanol on cAMP activation and renin production. At present, the role of distinct Cx molecule in mediating hemichannel activity and activating cAMP pathway in renin-secreting cells is unclear. Our preliminary experiment using siRNA against individual Cx revealed that downregulation of Cx43 alone appeared to be enough to influence CREB phosphorylation (Supplementary Figure S1). As a predominant Cx expressed in many cell types, Cx43-forming hemichannels might play a major role in channel permeability in As4.1 cells. Further characterization of Cx molecules involved in the control of hemichannel activity and the cAMP pathway in the renin-secreting juxtaglomerular cells, using strategies such as specific siRNA or connexin mimetic peptide, may lead to the development of more effective approaches in the control of renin secretion in vivo.

Of note, other than its action on Cx channels, heptanol used in this study also has other biological effects. Given that the effects achieved by heptanol were similarly obtained by siRNAs and several structurally different Cx channel inhibitors, it is conceivable that the observed effects were most likely due to its action on Cx channels.

Apart from elimination of intra- or extra-cellular Ca^2+^, hemichannels are also activated by Cx mutations, depolarization of the membrane potential, mechanic strain, and hypoxia, as well as by changes of cellular redox status [16,22,44]. It is presently unclear whether hemichannel opening initiated by other activators also similarly affects the cAMP signaling pathway. More detailed investigation in this direction may be needed in the future.

It is also interesting to mention that Cx hemichannels are reported to be activated by cAMP itself and cAMP-elevating agents, such as nitric oxide [45,46,47,48], the participation of hemichannels in mediating the activation of the cAMP signaling pathway suggests that there may exist a positive reciprocal regulatory loop between Cxs and cAMP in the amplification and sustainment of the cAMP signaling pathway. This idea also needs testing in the future studies.

In the last several years, great attention has been paid on the role of GJs in the control of renin. Several lines of evidences indicate that coupling through Cx40 channels is required for regulative mechanisms of renin expression and secretion as well as the correct localization of renin-expressing cells. Disruption of Cx40-mediated intercellular signaling leads to loss of pressure control of renin secretion and development of hypertension [19,21,49,50]. Our current findings suggest that Cxs might regulate renin through multiple mechanisms and toward the opposite direction. Depending on the context, Cx-forming channels might also contribute to renin synthesis and secretion. Apart from the stimulation of renin synthesis and secretion, cAMP-signaling pathway also participates in the regulation of a wide spectrum of cell processes, including cell migration, constriction, proliferation and differentiation. It is conceivable that hemichannels might be implicated in many pathophysiological situations and serve as potential targets for therapeutic intervention. Future studies will be focused on unraveling the underlying mechanisms behind this regulatory effect of hemichannels. Progress in this direction may provide further insight into gap junction physiology in the cardiovascular system and other organ systems as well.

In summary, our study characterizes hemichannel opening as an indispensable event for the Ca^2+^-elicited activation of the cAMP signaling pathway in renin-secreting cells. Our finding thus provides novel mechanistic insights into the regulatory effects of low Ca^2+^ on renin production and highlights the potential importance of Cx hemichannels in the cardiovascular system.

## 4. Materials and Methods

### 4.1. Reagents

Anti-Cx45 was from Santa Cruz (#sc-374354, Santa Cruz, CA, USA). Anti-phospho-cAMP response element-binding protein (CREB; Ser133; #11052) and anti-CREB (#21052-2) were purchased from Signalway Antibody (Pearland, TX, USA). Anti-phospho-vasodilator-stimulated phosphoprotein (anti-p-VASP, Ser157; #AB3839) and anti-Cx40 antibody (#AB1726) were obtained from Calbiochem (Darmstadt, Germany). Anti-renin antibody was bought from AnaSpec (#54371; San Jose, CA, USA) and also gifted by Prof. T lnagami (Department of Biochemistry, Vanderbilt University). FITC-conjugated swine anti-rabbit immunoglobulin (#F0205) was purchased from DAKO (Glostoup, Denmark). Anti-Cx43 (#C6219), anti-β-actin (#A3854), heptanol, lucifer yellow (LY), trypsin and all other chemicals were obtained from Sigma-Aldrich Japan (Tokyo, Japan).

### 4.2. Cell Culture

The renin-expressing As4.1 cell line was obtained from the American Type Culture Collection (ATCC No. CRL2193). For maintenance and passage, cells were cultured at 37 °C in RPMI-1640 with L-glutamine, phenol red and HEPES medium (Wako, Tokyo, Japan) supplemented with 5% fetal bovine serum (FBS). For comparison of cell responses between normal Ca^2+^ and Ca^2+^-free situations, cells were exposed to Ca^2+^-free DMEM (Gibco-BRL, Catalogue number #21068) with or without supplementation of 1.8 mM Ca^2+^.

### 4.3. Immunocytochemical Analysis

Immunocytochemical staining for Cx40, 43 and 45 was performed as previously reported [7,29]. In brief, cultured As4.1 cells were fixed in 2% paraformaldehyde, permeabilized with 1% Triton X-100, and incubated overnight with anti-Cx40, Cx43 and Cx45 antibody (diluted 1:100 in 1% FBS in PBS; 4 °C). After rinsing with PBS, the appropriate secondary antibody was added for 2 h at room temperature. The chamber slides were covered with Tris-buffered moviol (pH 8.6), and microscopy was performed with an Olympus BX50 microscope with a 40 × Planapo and 570 nm emission filter. Immuofluorescent images were captured using a CCD camera attached to the microscope.

### 4.4. Evaluation of GJIC by Microinjection of LY

GJIC was assessed by transfer of the membrane-impermeable fluorescent dye, LY after single cell microinjection using an automated microinjection system (Zeiss Oberkochen, Jena, Germany), as described previously [7].

### 4.5. Dye Uptake Assay

Hemichannel permeability was evaluated by the cellular uptake of LY as described previously [22,29,30,31]. As4.1 cells were exposed to Ca^2+^-free medium in the presence or absence of 0.05% LY for 20 min. The cells were then rinsed and fixed with 3% paraformaldehyde. Immunofluorescent images were captured using a CCD camera attached to an Olympus IX71 microscope (Tokyo, Japan).

### 4.6. ATP Measurement

ATP was measured using a luciferin/luciferase bioluminescence assay kit (Molecular Probes). The intensity of chemiluminescent signal was determined by a luminometer (Gene Light 55; Microtech Nition, Chiba, Japan) as described previously [7,22,23,29,30,31].

### 4.7. Western Blot Analysis

Western blot was performed using an enhanced chemiluminescence system [22,23,29,30,31]. Briefly, equal amounts of extracted cellular proteins were separated by 10% SDS–polyacrylamide gels and electrotransferred onto polyvinylidene difluoride membranes. After blocking with 3% BSA in PBS, the membranes were incubated with primary antibody. After washing with PBS-0.1% Tween 20, filters were probed with horseradish peroxidase–conjugated sheep anti-rabbit IgG or rabbit anti-mouse IgG (Cell Signaling; Beverly, MA, USA). Immunoreactivity was detected by an enhanced chemiluminescence system (Amersham Biosciences, Buckinghamshire, UK). The chemiluminescent signal was captured with a Fujifilm luminescent image LAS-4000 analyzer (Fujifilm, Tokyo, Japan). Densitometric quantitation of the blots was performed using the Scion Image software (Scion Corporation).

### 4.8. Transient Transfection of As4.1 Cells with siRNA

Cells were transiently transfected with siRNA specifically targeting against Cx37 (Mm_Gja4_1 FlexiTube siRNA), Cx40 (Mm_Gja5_1 FlexiTube siRNA), Cx43 (Mm_Gja 1_2 HP siRNA) and Cx45 (Mm_Gja7_2 FlexiTube siRNA, Qiagen, Japan) or a negative control siRNA (AllStars Negative Control siRNA) at a final concentration of 20 nM using Hyperfect transfection reagent for 24 h [22]. After transfection, cells were left untreated or exposed to Ca^2+^-free medium for the indicated time. Cellular protein was extracted and subjected to western blot analysis of the targeted proteins.

### 4.9. Statistical Analysis

Values are expressed as mean ± SE. Comparison of two populations was performed using a Student’s *t*-test. The analysis was performed using SigmaStat statistical software (Jandel Scientific, CA, USA). *p* < 0.05 was considered to be a statistically significant difference.

## Supplementary Materials

The following are available online at https://www.mdpi.com/1422-0067/21/12/4462/s1, Figure S1: Effects of treatment of As4.1 cells with siRNA against distinct Cx molecule on Ca^2+^ deprivation-induced phosphorylation of CREB. As4.1 cells were treated with the indicated siRNA against Cx37, 40, 43 and 45 for 24 h. After that, they were exposed to Ca^2+^-free medium for 30 min. Cellular protein was extracted and subjected to Western blot analysis for the effectiveness of Cx43 siRNA in downregulation of Cx43 protein level (A) and phosphorylated CREB (B). Note the elevation of p-CREB following removal of extracellular Ca^2+^ and its suppression by Cx43 siRNA.

## Abbreviations

ATP	adenosine triphosphate
Ca^2+^	calcium
CREB	cAMP response element binding protein
Cx	connexin
GJ	gap junction
JG	juxtaglomerular
NAD^+^	nicotinamide adenine dinucleotide
NRK	normal rat kidney
PGE2	prostaglandin E2
PKA	protein kinase A
LY	lucifer yellow
VASP	vasodilator-stimulated phosphoprotein
