# Supplementary material for: Connexin Hemichannels Contribute to the Activation of cAMP Signaling Pathway and Renin Production
Source: Int J Mol Sci. 2020 Jun 23;21(12):4462. doi: 10.3390/ijms21124462 (PMC7353028; doi:10.3390/ijms21124462)
Supplement: Supplementary file 1 [file ijms-21-04462-s001.pdf]

# Supplementary Figure 1

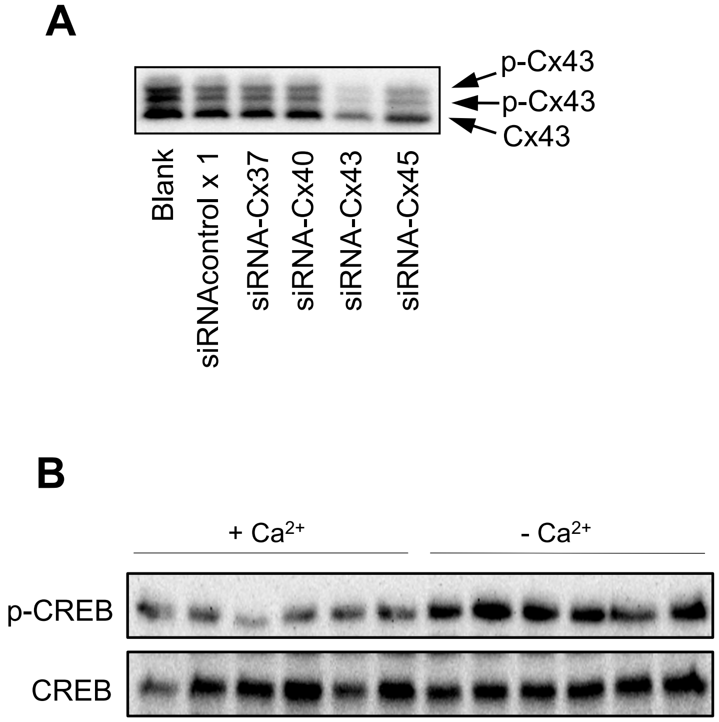


**Supplementary Figure 1. Effects of treatment of As4.1 cells with siRNA against distinct Cx molecule on calcium deprivation-induced phosphorylation of CREB.** As4.1 cells were treated with the indicated siRNA against Cx37, 40, 43 and 45 for 24 h. After that, they were exposed to calcium-free medium for 30 min. Cellular protein was extracted and subjected to Western blot analysis for the effectiveness of Cx43 siRNA in downregulation of Cx43 protein level (A) and phosphorylated CREB (B). Note the elevation of p-CREB following removal of extracellular calcium and its suppression by Cx43 siRNA.
